# Real-time topic-aware influence maximization using preprocessing

**DOI:** 10.1186/s40649-016-0033-z

**Published:** 2016-11-10

**Authors:** Wei Chen, Tian Lin, Cheng Yang

**Affiliations:** 1 Microsoft Research, No. 5 Danling Street, 100080 Beijing, China; 2 Institute for Advanced Study, Tsinghua University, No. 1 Tsinghua Yuan, 100084 Beijing, China; 3 Department of Computer Science and Technology, Tsinghua University, No. 1 Tsinghua Yuan, 100084 Beijing, China

**Keywords:** Influence maximization, Topic-aware influence modeling, Information diffusion

## Abstract

**Background:**

Influence maximization is the task of finding a set of seed nodes in a social network such that the influence spread of these seed nodes based on certain influence diffusion model is maximized. Topic-aware influence diffusion models have been recently proposed to address the issue that influence between a pair of users are often topic-dependent and information, ideas, innovations etc. being propagated in networks are typically mixtures of topics.

**Methods:**

In this paper, we focus on the topic-aware influence maximization task. In particular, we study preprocessing methods to avoid redoing influence maximization for each mixture from scratch.

**Results:**

We explore two preprocessing algorithms with theoretical justifications.

**Conclusions:**

Our empirical results on data obtained in a couple of existing studies demonstrate that one of our algorithms stands out as a strong candidate providing microsecond online response time and competitive influence spread, with reasonable preprocessing effort.

**Electronic supplementary material:**

The online version of this article (doi:10.1186/s40649-016-0033-z) contains supplementary material, which is available to authorized users.

## Background

In a social network, information, ideas, rumors, and innovations can be propagated to a large number of people because of the social influence between the connected peers in the network. *Influence maximization* is the task of finding a set of *seed nodes* in a social network such that the influence propagated from the seed nodes can reach the largest number of people in the network. More technically, a social network is modeled as a graph with nodes representing individuals and directed edges representing influence relationships. The network is associated with a stochastic diffusion model (such as independent cascade model and linear threshold model [1]) characterizing the influence propagation dynamics starting from the seed nodes. Influence maximization is to find a set of *k* seed nodes in the network such that the *influence spread*, defined as the expected number of nodes influenced (or activated) through influence diffusion starting from the seed nodes, is maximized [1, 2].

Influence maximization has a wide range of applications including viral marketing [1, 3, 4], information monitoring and outbreak detection [5], competitive viral marketing and rumor control [6, 7], or even text summarization [8] (by modeling a word influence network). As a result, influence maximization has been extensively studied in the past decade. Research directions include improvements in the efficiency and scalability of influence maximization algorithms [9–11], extensions to other diffusion models and optimization problems [6, 7, 12], and influence model learning from real-world data [13–15].

Most of these works treat diffusions of all information, rumors, ideas, etc. (collectively referred as *items* in this paper) as following the same model with a single set of parameters. In reality, however, influence between a pair of friends may differ depending on the topic. For example, one may be more influential to the other on high-tech gadgets, while the other is more influential on fashion topics, or one researcher is more influential on data mining topics to her peers but less influential on algorithm and theory topics. Recently, Barbieri et al. [16] propose the topic-aware independent cascade (TIC) and linear threshold (TLT) models, in which a diffusion item is a mixture of topics and influence parameters for each item are also mixtures of parameters for individual topics. They provide learning methods to learn influence parameters in the topic-aware models from real-world data. Such topic-mixing models require new thinking in terms of the influence maximization task, which is what we address in this paper.

In this paper, we adopt the models proposed in [16] and study efficient topic-aware influence maximization schemes. One can still apply topic-oblivious influence maximization algorithms in online processing of every diffusion item, but it may not be efficient when there are a large number of items with different topic mixtures or real-time responses are required. Thus, we focus on preprocessing individual topic influence such that when a diffusion item with certain topic mixture comes, the online processing of finding the seed set is fast. To do so, our first step is to collect two datasets in the past studies with available topic-aware influence analysis results on real networks and investigate their properties pertaining to our preprocessing purpose ("Data observation" section). Our data analysis shows that in one network users and their relationships are largely separated by different topics while in the other network they have significant overlaps on different topics. Even with this difference, a common property we find is that in both datasets most top seeds for a topic mixture come from top seeds of the constituent topics, which matches our intuition that influential individuals for a mixed item are usually influential in at least one topic category.

Motivated by our findings from the data analysis, we explore two preprocessing based algorithms ("Preprocessing based algorithms" section). The first algorithm, *Best Topic Selection* (BTS), minimizes online processing by simply using a seed set for one of the constituent topics. Even for such a simple algorithm, we are able to provide a theoretical approximation ratio (when a certain property holds), and thus BTS serves as a baseline for preprocessing algorithms. The second algorithm, *Marginal Influence Sort* (MIS), further uses pre-computed marginal influence of seeds on each topic to avoid slow greedy computation. We provide a theoretical justification showing that MIS can be as good as the offline greedy algorithm when nodes are fully separated by topics.

We then conduct experimental evaluations of these algorithms and comparing them with both the greedy algorithm and a state-of-the-art heuristic algorithm PMIA [10], on the two datasets used in data analysis as well as a third dataset for testing scalability ("Experiments" section). From our results, we see that MIS algorithm stands out as the best candidate for preprocessing based real-time influence maximization: it finishes online processing within a few microseconds and its influence spread either matches or is very close to that of the greedy algorithm.

Our work, together with a recent independent work [17], is one of the first that study topic-aware influence maximization with focus on preprocessing. Comparing to [17], our contributions include: (a) we include data analysis on two real-world datasets with learned influence parameters, which shows different topical influence properties and motivates our algorithm design; (b) we provide theoretical justifications to our algorithms; (c) the use of marginal influence of seeds in individual topics in MIS is novel, and is complementary to the approach in [17]; (d) even though MIS is quite simple, it achieves competitive influence spread within microseconds of online processing time rather than milliseconds needed in [17].

## Preliminaries

In this section, we introduce the background and problem definition on the topic-aware influence diffusion models. We focus on the independent cascade model [1] for ease of presentation, but our results also hold for other models parameterized with edge parameters such as the linear threshold model [1].

### Independent cascade model

We consider a social network as a directed graph $G=(V,E)$, where each node in *V* represents a user, and each edge in *E* represents the relationship between two users. For every edge $(u, v) \in E$, denote its *influence probability* as $p(u, v) \in [0, 1]$, and for all $(u, v) \notin E$ or $u = v$, we assume $p (u, v) = 0$.

The *independent cascade* (IC) model, defined in [1], captures the stochastic process of contagion in discrete time. Initially at time step $t=0$, a set of nodes $S \subseteq V$ called *seed nodes* are activated. At any time $t \ge 1$, if node *u* is activated at time $t-1$, it has one chance of activating each of its inactive outgoing neighbor *v* with probability *p*(*u*, *v*). A node stays active after it is activated. This process stops when no more nodes are activated. We define *influence spread* of seed set *S* under influence probability function *p*, denoted $\sigma (S, p)$, as the expected number of active nodes after the diffusion process ends. As shown in [1], for any fixed *p*, $\sigma (S, p)$ is monotone [i.e., $\sigma (S, p) \le \sigma (T, p)$ for any $S \subseteq T$] and submodular [i.e., $\sigma (S \cup \{ v \}, p) - \sigma (S, p) \ge \sigma (T \cup \{ v \}, p) - \sigma (T, p)$ for any $S \subseteq T$ and $v \in V$] on its seed set parameter. The next lemma further shows that for any fixed *S*, $\sigma (S, p)$ is monotone in *p*. For two influence probability functions *p* and $p'$ on graph $G=(V,E)$, we denote $p \le p'$ if for any $(u,v)\in E$, $p(u,v) \le p'(u,v)$. We say that influence spread function $\sigma (S, p)$ is *monotone in p* if for any $p\le p'$, we have $\sigma (S,p) \le \sigma (S,p')$.

#### **Lemma 1**

*For any fixed seed set* $S \subseteq V$, $\sigma (S, p)$ *is monotone in p*.

#### Proof sketch

We use the following coupling method. For any edge $(u,v)\in E$, we select a number *x*(*u*, *v*) uniformly at random in [0, 1]. Then for any influence probability function *p*, we select edge (*u*, *v*) as a *live edge* if $x(u,v) \le p(u,v)$ and otherwise it is a *blocked edge*. All live edges form a random *live-edge graph* $G_L(p)$. One can verify that $\sigma (S,p)$ is the expected value of the size of node set reachable from *S* in random graph $G_L(p)$. Moreover, for *p* and $p'$ such that $p \le p'$, one can verify that after fixing the random numbers $x(u,v)'s$, live-edge graph $G_L(p)$ is a subgraph of live-edge graph $G_L(p')$, and thus nodes reachable from *S* in $G_L(p)$ must be also reachable from *S* in $G_L(p')$. This implies that $\sigma (S,p) \le \sigma (S,p').$ $\hfill\square$

We remark that using a similar idea as above we could show that influence spread in the linear threshold (LT) model [1] is also monotone in the edge weight parameter.
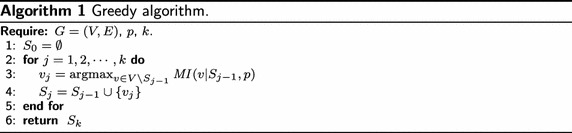


### Influence maximization

Given a graph $G=(V,E)$, an influence probability function *p*, and a budget *k*, *influence maximization* is the task of selecting at most *k* seed nodes such that the influence spread is maximized, i.e., finding set $S^{*}=S^{*}(k,p)$ such that

$$\begin{aligned} S^{*}(k,p) = \mathop {\arg \max }\limits _{S \subseteq V,\left| S \right| \le k} \sigma (S, p). \end{aligned}$$

In [1], Kempe et al. show that the influence maximization problem is NP-hard in both the IC model and the LT model. They propose the greedy approach for influence maximization, as shown in Algorithm 1. Given influence probability function *p*, the *marginal influence (MI)* of a node *v* under seed set *S* is defined as ${ MI}(v | S, p) = \sigma (S \cup \{v\}, p)-\sigma (S, p)$, for any $v\in V$. The greedy algorithm selects *k* seeds in *k* iterations, and in the *j*-th iteration it selects a node $v_j$ with the largest marginal influence under the current seed set $S_{j-1}$ and adds $v_j$ into $S_{j-1}$ to obtain $S_j$. Kempe et al. use Monte Carlo simulations to obtain accurate estimates on marginal influence ${ MI}(v | S, p)$, and later Chen et al. show that indeed exact computation of influence spread $\sigma (S,p)$ or marginal influence ${ MI}(v | S, p)$ is #P-hard [10]. The monotonicity and submodularity of $\sigma (S,p)$ in *S* guarantees that the greedy algorithm selects a seed set with approximation ratio $1-\frac{1}{e} - \varepsilon$, that is, it returns a seed set $S^{g}= S^{g}(k, p)$ such that

$$\begin{aligned} \sigma (S^{g}, p) \ge \left( 1-\frac{1}{e} - \varepsilon \right) \sigma (S^{*},p), \end{aligned}$$

for any small $\varepsilon > 0$, where $\varepsilon$ accommodates the inaccuracy in Monte Carlo estimations.

### Topic-aware independent cascade model and topic-aware influence maximization

Topic-aware independent cascade (TIC) model [16] is an extension of the IC model to incorporate topic mixtures in any diffusion item. Suppose there are *d* base topics, and we use set notation $[d] = \{1,2,\ldots ,d\}$ to denote topic $1,2, \ldots , d$. We regard each diffusion item as a distribution of these topics. Thus, any item can be expressed as a vector $I=(\lambda _1, \lambda _2, \dots , \lambda _{d})$ where $\forall i \in [d]$, $\lambda _i \in [0,1]$ and $\sum _{i \in [d]} \lambda _i=1$. We also refer $(\lambda _1, \lambda _2, \dots , \lambda _{d})$ as a *topic mixture*. Given a directed social graph $G=(V,E)$, for any topic $i \in [d]$, influence probability on that topic is $p_i{:\,} V \times V \rightarrow [0,1]$, and for all $(u, v) \notin E$ or $u = v$, we assume $p_i(u, v) = 0$. In the TIC model, the influence probability function *p* for any diffusion item $I=(\lambda _1,\lambda _2,\dots ,\lambda _{d})$ is defined as $p(u,v) = \sum _{i \in [d]} \lambda _i {p_i}(u,v)$, for all $u,v \in V$ (or simply $p = \sum _{i \in [d]} \lambda _i {p_i}$). Then, the stochastic diffusion process and influence spread $\sigma (S, p)$ are exactly the same as defined in the IC model by using the influence probability *p* on edges.

Given a social graph *G*, base topics [*d*], influence probability function $p_i$ for each base topic *i*, a budget *k* and an item $I=(\lambda _1,\lambda _2,\dots ,\lambda _{d})$, the *topic-aware influence maximization* is the task of finding optimal seeds $S^{*}= S^{*}(k, p) \subseteq V$, where $p = \sum _{i \in [d]} \lambda _i {p_i}$, to maximize the influence spread, i.e.,

$$\begin{aligned} S^{*}= \mathop {\arg \max }\limits _{S \subseteq V,\left| S \right| \le k} \sigma (S, p). \end{aligned}$$

## Data observation

There are relatively few studies on topic-aware influence analysis. For our study, we are able to obtain datasets from two prior studies, one is on social movie rating network Flixster [16] and the other is on academic collaboration network Arnetminer [14]. In this section, we describe these two datasets, and present statistical observations on these datasets, which will help us in our algorithm design.

### Data description

We obtain two real-world datasets, Flixster and Arnetminer, which include influence analysis results from their respective raw data, from the authors of the prior studies [14, 16].

Flixster^1^ is an American social movie site for discovering new movies, learning about movies, and meeting others with similar tastes in movies. The raw data in Flixster dataset is the action traces of movie ratings of users. The Flixster network represents users as nodes, and two users *u* and *v* are connected by a directed edge (*u*, *v*) if they are friends both rating the same movie and *v* rates the movie shortly later after *u* does so. The network contains 29,357 nodes, 425,228 directed edges and 10 topics [16]. Barbieri et al. [16] use their proposed TIC model and apply maximum likelihood estimation method on the action traces to obtain influence probabilities on edges for all 10 topics. We found that there are a disproportionate number of edges with influence probabilities higher than 0.99, which is due to the lack of sufficient samplings of propagation events over these edges. We smoothen these influence probability values by changing all the probabilities larger than 0.99 to random numbers according to the probability distribution of all the probabilities smaller than 0.99. We also obtain 11,659 topic mixtures, and demonstrate the distribution of the number of topics in item mixtures in Table 1. We eliminate individual probabilities that are too weak ($\forall i\in [d], \,\lambda _i < 0.01$). In general, most items are on a single topic only, with some two-topic mixtures. Mixtures with three or four topics are already rare and there are no items with five or more topics.

**Table 1:** Distribution of topic numbers of mixture items in Flixster

Mixed topics	1	2	3	4	5
Samples	11,285	354	18	2	0
(%)	(96.79)	(3.04)	(0.15)	(0.02)	(0.00)

Arnetminer^2^ is a free online service used to index and search academic social networks. The Arnetminer network represents authors as nodes and two authors have an edge if they coauthored a paper. The raw data in the Arnetminer dataset is not the action traces but the topic distributions of all nodes and the network structure [14]. Tang et al. apply factor graph analysis to obtain influence probabilities on edges from node topic distributions and the network structure [14]. The resulting network contains 5114 nodes, 34,334 directed edges and 8 topics, and all 8 topics are related to computer science, such as data mining, machine learning, information retrieval, etc. Mixed items propagated in such academic networks could be ideas or papers from related topic mixtures, although there are no raw data of topic mixtures available in Arnetminer.

Table 2 provides statistics for the learned influence probabilities for every topic in Arnetminer and Flixster dataset. Column “nonzero” provides the number of edges having nonzero probabilities on the specific topic. Other columns are mean, standard deviation, 25-, 50-% (median), and 75-% of the probabilities among the nonzero entries. The basic statistics show similar behavior between the two datasets, such as mean probabilities are mostly between 0.1 and 0.2, standard deviations are mostly between 0.1 and 0.3, etc. Comparing among different topics, even though the means and other statistics are similar to one another, the number of nonzero edges may have up to tenfold difference. This indicates that some topics are more likely to propagate than others.

**Table 2:** Influence probability statistics

Topic	Nonzero	Mean	Std.	25%	50%	75%
(a) Arnetminer						
1	3355	0.175	0.230	0.023	0.075	0.229
2	13,331	0.093	0.154	0.010	0.031	0.100
3	3821	0.158	0.214	0.020	0.065	0.201
4	1537	0.217	0.243	0.038	0.120	0.316
5	2468	0.197	0.262	0.018	0.080	0.266
6	1236	0.240	0.273	0.034	0.122	0.353
7	4439	0.145	0.222	0.011	0.046	0.177
8	3439	0.162	0.220	0.022	0.069	0.201
(b) Flixster						
1	54,032	0.173	0.215	1.00E−04	0.086	0.264
2	84,322	0.172	0.227	4.36E−05	0.067	0.260
3	231,807	0.089	0.146	1.18E−04	0.024	0.112
4	35,394	0.162	0.226	6.78E−03	0.050	0.250
5	118,125	0.097	0.141	2.45E−03	0.037	0.131
6	37,489	0.090	0.142	6.85E−03	0.033	0.100
7	84,716	0.166	0.230	3.12E−05	0.050	0.250
8	149,140	0.097	0.145	9.01E−04	0.036	0.131
9	152,181	0.103	0.158	2.14E−04	0.032	0.140
10	139,335	0.159	0.235	3.27E−05	0.029	0.250

### Topic separation on edges and nodes

For the two datasets, we would like to investigate how different topics overlap on edges and nodes. To do so, we define the following coefficients to characterize the properties of a social graph.

Given threshold $\theta \ge 0$, for every topic *i*, denote edge set $\tau _i(\theta ) = \{ (u,v) \in E\,|\, {p_i}(u,v) > \theta \}$, and node set $\nu _i(\theta ) = \{v\in V \,|\, \sum _{u:(v,u)\in E} p_i(v,u)+ \sum _{u:(u,v)\in E} p_i(u,v) > \theta \}$. For topics *i* and *j*, we define *edge overlap coefficient* as $R^{E}_{ij}(\theta ) = \frac{|\tau _i(\theta ) \cap \tau _j(\theta )|}{\min \{|\tau _i(\theta )|, |\tau _j(\theta )|\}}$, and *node overlap coefficient* as $R^{V}_{ij}(\theta ) = \frac{|\nu _i(\theta ) \cap \nu _j(\theta )|}{\min \{|\nu _i(\theta )|, |\nu _j(\theta )|\}}$. If $\theta$ is small and the overlap coefficient is small, it means that the two topics are fairly separated in the network. In particular, we say that the network is *fully separable* for topics *i* and *j* if $R^{V}_{ij}(0) = 0$, and it is fully separable for all topics if $R^{V}_{ij}(0) = 0$ for any pair *i* and *j* with $i\ne j$. Then we apply the above coefficients to the Flixster and Arnetminer datasets.

**Table 3:** Edge and node overlap coefficients on Arnetminer

–	0.017	0.002	0.000	0.005	0.006	0.000	0.022
*0.068*	–	0.001	0.004	0.001	0.001	0.002	0.000
*0.018*	*0.014*	–	0.000	0.000	0.001	0.000	0.000
*0.002*	*0.029*	*0.000*	–	0.000	0.011	0.017	0.000
*0.025*	*0.005*	*0.005*	*0.000*	–	0.000	0.000	0.015
*0.054*	*0.049*	*0.049*	*0.011*	*0.000*	–	0.009	0.001
*0.006*	*0.025*	*0.003*	*0.017*	*0.007*	*0.063*	–	0.000
*0.108*	*0.001*	*0.008*	*0.000*	*0.079*	*0.011*	*0.004*	–

The upper triangle represents edge overlap coefficient when $\theta =0.1$. The entry on row *i*, column *j* represents $R^{E}_{ij}(0.1)$; the lower italic triangle represents node overlap coefficient when $\theta =0.1$. The entry on row *i*, column *j* represents $R^{V}_{ij}(0.1)$

Table 3 shows the edge and node overlap coefficients with threshold $\theta =0.1$ for every pair of topics in the Arnetminer dataset. Correlating with Table 2a, we see that $\theta =0.1$ is around the mean value for all topics. Thus it is a reasonably small value especially for the node overlap coefficients, which is about aggregated probability of all edges incident to a node. A clear indication in Table 3 is that topic overlap on both edges and nodes are very small in Arnetminer, with most node overlap coefficients less than $5\%$. We believe that this is because in academic collaboration network, most researchers work on one specific research area, and only a small number of researchers work across different research areas.

**Table 4:** Edge overlap coefficients on Flixster

–	0.33	0.49	0.27	0.36	0.35	0.35	0.42	0.43	0.39
*0.22*	–	0.48	0.33	0.31	0.41	0.31	0.36	0.38	0.39
*0.28*	*0.26*	–	0.46	0.50	0.48	0.55	0.50	0.57	0.52
*0.15*	*0.19*	*0.22*	–	0.33	0.25	0.31	0.37	0.38	0.38
*0.20*	*0.25*	*0.34*	*0.13*	–	0.52	0.30	0.46	0.45	0.37
*0.23*	*0.29*	*0.28*	*0.16*	*0.31*	–	0.36	0.50	0.47	0.38
*0.25*	*0.21*	*0.34*	*0.18*	*0.24*	*0.25*	–	0.37	0.43	0.46
*0.21*	*0.24*	*0.38*	*0.15*	*0.31*	*0.29*	*0.25*	–	0.44	0.37
*0.24*	*0.24*	*0.44*	*0.17*	*0.32*	*0.28*	*0.29*	*0.35*	–	0.42
*0.28*	*0.27*	*0.47*	*0.23*	*0.29*	*0.26*	*0.35*	*0.32*	*0.37*	–

The upper triangle represents edge overlap coefficient when $\theta =0.1$. The entry on row *i*, column *j* represents $R^{E}_{ij}(0.1)$; the lower italic triangle represents edge overlap coefficient when $\theta =0.3$. The entry on row *i*, column *j* represents $R^{E}_{ij}(0.3)$

Tables 4 and 5 show the edge and node overlap coefficients for the Flixster dataset. Different from the Arnetminer dataset, both edges and nodes have significant overlaps. For edge overlaps, even with threshold $\theta =0.3$, all topic pairs have edge overlap between 15 and $40\%$. For node overlap, we test the threshold for both 0.5–5, but the overlap coefficients do not significantly change: at $\theta =5$, most pairs still have above $60\%$ and up to $89\%$ overlap. We think that this could be explained by the nature of Flixster, which is a movie rating site. Most users are interested in multiple categories of movies, and their influence to their friends are also likely to be across multiple categories. It is interesting to see that, even though the per-topic statistics between Arnetminer and Flixster are similar, they show quite different cross-topic overlap behaviors, which can be explained by the nature of the networks. This could be an independent research topic for further investigations on the influence behaviors among different topics.

**Table 5:** Node overlap coefficients on Flixster

–	0.79	0.91	0.68	0.76	0.81	0.77	0.83	0.85	0.87
*0.69*	–	0.88	0.82	0.76	0.88	0.75	0.74	0.77	0.84
*0.83*	*0.64*	–	0.93	0.92	0.95	0.91	0.92	0.91	0.87
*0.53*	*0.67*	*0.75*	–	0.77	0.63	0.78	0.83	0.85	0.89
*0.58*	*0.70*	*0.87*	*0.50*	–	0.90	0.73	0.84	0.85	0.85
*0.76*	*0.83*	*0.86*	*0.46*	*0.91*	–	0.86	0.93	0.92	0.91
*0.71*	*0.53*	*0.72*	*0.62*	*0.72*	*0.78*	–	0.77	0.81	0.88
*0.72*	*0.57*	*0.82*	*0.60*	*0.85*	*0.89*	*0.59*	–	0.83	0.84
*0.74*	*0.53*	*0.84*	*0.62*	*0.82*	*0.89*	*0.63*	*0.73*	–	0.83
*0.89*	*0.74*	*0.81*	*0.83*	*0.88*	*0.89*	*0.82*	*0.82*	*0.84*	–

The upper triangle represents node overlap coefficient when $\theta =0.5$. The entry on row *i*, column *j* represents $R^{V}_{ij}(0.5)$; the lower italic triangle represents node overlap coefficient when $\theta =5.0$. The entry on row *i*, column *j* represents $R^{V}_{ij}(5.0)$

Table 6 summarizes the edge and node overlap coefficient statistics among all pairs of topics for the two datasets. We can see that Arnetminer network has fairly separate topics on both nodes and edges, while Flixter network have significant topic overlaps. This may be explained by that in an academic network most researchers only work in one research area, but in a movie network many users are interested in more than one type of movies. Therefore, our first observation is:

**Table 6:** Overlap coefficient statistics for all topic pairs

	Min	Mean	Max
Arnetminer: $R^{E}_{ij}(0.1)$	0	0.0041	0.022
Arnetminer: $R^{V}_{ij}(0.1)$	0	0.0236	0.108
Flixster: $R^{E}_{ij}(0.1)$	0.25	0.4058	0.57
Flixster: $R^{E}_{ij}(0.3)$	0.13	0.2662	0.47
Flixster: $R^{V}_{ij}(0.5)$	0.63	0.836	0.95
Flixster: $R^{V}_{ij}(5.0)$	0.46	0.734	0.91

#### Observation 1

Topic separation in terms of influence probabilities is network dependent. In the Arnetminer network, topics are mostly separated among different edges and nodes in the network, while in the Flixster network there are significant overlaps on topics among nodes and edges.

### Sources of seeds in the mixture

Our second observation is more directly related to influence maximization. We would like to see if seeds selected by the greedy algorithm for a topic mixture are likely coming from top seeds for each individual topic. Intuitively, it seems reasonable to assume that top influencers for a topic mixture are likely from top influencers in their constituent topics.

To check the source of seeds, we randomly generate 50 mixtures of two topics for both Arnetminer and Flixster, and use the greedy algorithm to select seeds for the mixture and the constituent topics. We then check the percentage of seeds in the mixture that is also in the constituent topics. Table 7 shows our test results (Flixster (Dirhilect) is the result using a Dirichlet distribution to generate topic mixtures; see "Experiments" section for more details). Our observation below matches our intuition:

**Table 7:** Percentage of seeds in topic mixture that are also seeds of constituent topics

	Arnetminer	Flixster (random)	Flixster (Dirichlet)
Seeds overlap (%)	94.80	81.16	85.24

#### Observation 2

Most seeds for topic mixtures come from the seeds of constituent topics, in both Arnetminer and Flixster networks.

For Arnetminer, it is likely due to the topic separation as observed in Table 3. For Flixster, even though topics have significant overlaps, these overlaps may result in many shared seeds between topics, which would also contribute as top seeds for topic mixtures.

## Preprocessing based algorithms

Topic-aware influence maximization can be solved by using existing influence maximization algorithms such as the ones in [1, 10]: when a query on an item $I = (\lambda _1, \lambda _2, \ldots , \lambda _d)$ comes, the algorithm first computes the mixed influence probability function $p = \sum _j \lambda _j p_j$, and then applies existing algorithms using parameter *p*. This, however, means that for each topic mixture influence maximization has to be carried out from scratch, which could be inefficient in large-scale networks.
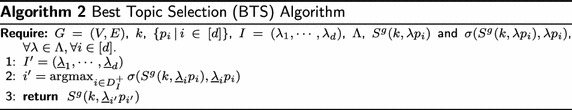


In this section, motivated by observations made in "Data observation" section, we introduce two preprocessing based algorithms that cover different design choices. The first algorithm Best Topic Selection focuses on minimizing online processing time, and the second one MIS uses pre-computed marginal influence to achieve both fast online processing and competitive influence spread. For convenience, we consider the budget *k* as fixed in our algorithms, but we could extend the algorithms to consider multiple *k* values in preprocessing.

### Best topic selection (BTS) algorithm

The idea of our first algorithm is to minimize online processing by simply selecting a seed set for one of the constituent topics in the topic mixture that has the best influence performance, and thus we call it Best Topic Selection (BTS) algorithm. More specifically, given an item $I = (\lambda _1, \lambda _2, \ldots , \lambda _{d})$, if we have pre-computed the seed set $S^{g}_i = S^{g}(k, \lambda p_i)$ via the greedy algorithm for each topic *i*, then we would simply use the seed set $S^{g}_{i'}$ that gives the best influence spread, i.e., $i' = \mathop {\arg \max }\nolimits _{i \in \left[ d \right] } \sigma (S^{g}_i, \lambda _i p_i)$. However, in the preprocessing stage, the topic mixture $(\lambda _1, \lambda _2, \ldots , \lambda _{d})$ is not guaranteed to be pre-computed exactly. To deal with this issue, we pre-compute influence spread for a number of landmark points for each topic, and use rounding method in online processing to complete seed selection, as we explain in more detail now.

#### *Preprocess stage*

Denote constant set $\Lambda = \{\lambda ^{c}_0, \lambda ^{c}_1, \lambda ^{c}_2, \ldots , \lambda ^{c}_m\}$ as a set of *landmarks*, where $0 = \lambda ^{c}_0< \lambda ^{c}_1< \cdots < \lambda ^{c}_m = 1$. For each $\lambda \in \Lambda$ and each topic $i \in [d]$, we pre-compute $S^{g}(k, \lambda p_i)$ and $\sigma (S^{g}(k, \lambda p_i), \lambda p_i)$ in the preprocessing stage, and store these values for online processing. In our experiments, we use uniformly selected landmarks and show that they are good enough for influence maximization. More sophisticated landmark selection method may be applied, such as the machine learning based method in [17].

#### *Online stage*

We define two rounding notations that return one of the neighboring landmarks in $\Lambda = \{\lambda ^{c}_0, \lambda ^{c}_1, \ldots , \lambda ^{c}_m\}$: for any $\lambda \in [0,1]$, $\underline{\lambda }$ is denoted as rounding $\lambda$ down to $\lambda ^{c}_j$ where $\lambda ^{c}_j \le \lambda < \lambda ^{c}_{j+1}$ and $\lambda ^{c}_j, \lambda ^{c}_{j+1} \in \Lambda$, and $\overline{\lambda }$ as rounding up to $\lambda ^{c}_{j+1}$ where $\lambda ^{c}_j < \lambda \le \lambda ^{c}_{j+1}$ and $\lambda ^{c}_j, \lambda ^{c}_{j+1} \in \Lambda$. Given $I = (\lambda _1, \lambda _2, \ldots , \lambda _{d})$, let $D^+_I = \{i\in [d] \,|\, \lambda _i > 0 \}$. With the pre-computed $S^{g}(k, \lambda p_i)$ and $\sigma (S^{g}(k, \lambda p_i), \lambda p_i)$ for every $\lambda \in \Lambda$ and every topic *i*, the BTS algorithm is given in Algorithm 2. The algorithm basically rounds down the mixing coefficient on every topic to $(\underline{\lambda }_1, \ldots , \underline{\lambda }_d)$, and then returns the seed set $S^{g}(k, \underline{\lambda }_{i'} p_{i'})$ that gives the largest influence spread at the round-down landmarks: $i' = \mathop {\arg \max }\nolimits _{i \in D_I^ + } \sigma (S^{g}(k, \underline{\lambda }_i p_{i}), \underline{\lambda }_i p_i)$.

BTS is rather simple since it directly outputs a seed set for one of the constituent topics. However, we show below that even such a simple scheme could provide a theoretical approximation guarantee (if the influence spread function is sub-additive as defined below). Thus, we use BTS as a baseline for preprocessing based algorithms.

We say that influence spread function $\sigma (S,p)$ is *c-sub-additive in p* for some constant *c* if for every set $S \subseteq V$ with $|S| \le k$ and every mixture $(\lambda _1, \lambda _2, \ldots , \lambda _d)$, $\sigma (S, \sum _{i \in D^+_I} \lambda _i p_i)$ $\le$ $c \sum _{i \in D^+_I} \sigma (S, \lambda _i p_i)$. The sub-additivity property above means that the influence spread of any seed set *S* in any topic mixture will not exceed constant times of the sum of the influence spread of the same seed set for each individual topic. It is easy to verify that, when the network is fully separable for all topic pairs, $\sigma (S,p)$ is 1-sub-additive. The only counterexample to the sub-additivity assumption that we could find is a tree structure where even layer edges are for one topic and odd layer edges are for another topic. Such structures are rather artificial, and we believe that for real networks the influence spread is *c*-sub-additive in *p* with a reasonably small constant *c*.

We define $\mu _{\max } = \max _{i \in [d], \lambda \in [0,1]} \frac{\sigma (S^{g}(k, \overline{\lambda }p_i), \overline{\lambda }p_i)}{\sigma (S^{g}(k, \underline{\lambda }p_i), \underline{\lambda }p_i)}$, which is a value controlled by preprocessing. A fine-grained landmark set $\Lambda$ could make $\mu _{\max }$ close to 1. The following Theorem 1 guarantees the theoretical approximation ratio of Algorithm 2.

##### **Theorem 1**

*If the influence spread function* $\sigma (S,p)$ *is c*-*sub-additive in* *p*,* Algorithm* 2* achieves* $\frac{1-e^{-1}}{c |D^+_I| \mu _{\max }}$ *approximation ratio for item* $I = (\lambda _1, \lambda _2, \ldots , \lambda _{d})$.

##### Proof

Denote $S^{*}= S^{*}(k, p)$, $\overline{S}^{*}_i = S^{*}(k, \overline{\lambda }_i p_i)$, $\overline{S}^{g}_i = S^{g}(k, \overline{\lambda }_i p_i)$ and $\underline{S}^{g}_i = S^{g}(k, \underline{\lambda }_i p_i)$. Since $\sigma (S, p)$ is monotone (Lemma 1) and *c*-sub-additive in *p*, it implies $\sigma (S^{*}, p) = \sigma (S^{*}, \sum _{i \in D^+_I} \lambda _i p_i) \le c \sum _{i \in D^+_I} \sigma (S^{*}, \lambda _i p_i)$ $\le$ $c \sum _{i \in D^+_I} \sigma (S^{*}, \overline{\lambda }_i p_i)$. From [1], we know $\sigma (S^{*}(k, p_0), p_0) \le \frac{1}{1-e^{-1}} \sigma (S^{g}(k, p_0), p_0)$ holds for any $p_0$ in Algorithm 1. Thus we have, for each $i \in D^+_I$, $\sigma (S^{*}, \overline{\lambda }_i p_i) \le \sigma (\overline{S}^{*}_i, \overline{\lambda }_i p_i) \le \frac{\sigma (\overline{S}^{g}_i, \overline{\lambda }_i p_i)}{1-e^{-1}} \le \frac{\mu _{\max } \cdot \sigma (\underline{S}^{g}_i, \underline{\lambda }_i p_i)}{1-e^{-1}}$. According to line 2 of Algorithm 2, $i'$ satisfies $\sigma (\underline{S}^{g}_{i'}, \underline{\lambda }_{i'} p_{i'}) = \max _{i \in D^+_I} \sigma (\underline{S}^{g}_i, \underline{\lambda }_i p_i)$, and $\sigma (\underline{S}^{g}_{i'}, \underline{\lambda }_{i'} p_{i'}) \le \sigma (\underline{S}^{g}_{i'}, \lambda _{i'} p_{i'})$. Thus, connecting all the inequalities, we have $\sigma (S^{*}, p)$ $\le$ $\frac{c |D^+_I| \mu _{\max }}{1-e^{-1}} \sigma (\underline{S}^{g}_{i'}, \lambda _{i'} p_{i'})$. Therefore, Algorithm 2 achieves approximation ratio of $\frac{1}{c |D^+_I| \mu _{\max }}(1-\frac{1}{e})$ under the sub-additive assumption. $\hfill\square$

The approximation ratio given in the theorem is a conservative bound for the worst case (e.g., a common setting may be $c=1$, $\mu _{\max }=1.5$, $|D^+_I|=2$). Tighter online bound in our experiment section based on [5] shows that Algorithm 2 performs much better than the worst case scenario.

### Marginal influence sort (MIS) algorithm

Our second algorithm derives the seed set from pre-computed seed set of constituent topics, which is based on Observation 2. Moreover, it uses marginal influence information pre-computed to help select seeds from different seed sets. Our idea is partially motivated from Observation 1, especially the observation on Arnetminer dataset, which shows that in some cases the network could be well separated among different topics. Intuitively, if nodes are separable among different topics, and each node *v* is only pertinent to one topic *i*, the marginal influence of *v* would not change much whether it is for a mixed item or the pure topic *i*. The following lemma makes this intuition precise for the extreme case of fully separable networks.

#### **Lemma 2**

*If a network is fully separable among all topics, then for any* $v \in V$ *and topic* $i \in [d]$ *such that* $\sigma (v, p_i) > 1$,* for any item* $I = (\lambda _1, \lambda _2, \dots , \lambda _{d})$,* for any seed set* $S \subseteq V$,* we have* ${ MI}(v | S, \lambda _i p_i) = { MI}(v | S, p)$,* where* $p = \sum _{j\in [d]} \lambda _j p_j.$

#### Proof sketch

Let $G_i=(V_i,E_i)$ be the subgraph of *G* generated by edges (*u*, *w*) such that $p_i(u,w) > 0$ and their incident nodes. It is easy to verify that when the network is fully separable among all topics, $G_i$ and $G_j$ are disconnected for any $i\ne j$. In this case, we have (a) for any node *v* and topic *i* such that $\sigma (v, p_i) > 1$, $v \in V_i$; (b) for any edge $(u,w)\in E_i$, $p(u,w) = \lambda _i p_i(u,w)$; and (c) $\sigma (S,p') = \sum _{j\in [d]} \sigma (S\cap V_j, p')$ for any $p'$. With the above property, a simple derivation following the definition of marginal influence will lead to ${ MI}(v | S, \lambda _i p_i) = { MI}(v | S, p).$ $\hfill  \square$

The above lemma suggests that we can use the marginal influence of a node on each topic when dealing with a mixture of topics. Algorithm MIS is based on this idea.

#### *Preprocess stage*

Recall the detail of Algorithm 1, given any fixed probability *p* and budget *k*, for each iteration $$j = 1,2,\ldots , k$$, it calculates $$v_j$$ to maximize marginal influence $${ MI}(v_j | S_{j-1}, p)$$ and let $$S_j = S_{j-1} \cup \{ v_j \}$$ every time, and output $$S^{g}(k, p) = S_k$$ as seeds. Let $${ MI}^{g}(v_j, p) = { MI}(v_j | S_{j-1}, p)$$, if $$v_j \in S^{g}(k, p)$$, and 0 otherwise. $${ MI}^{g}(v_j, p) $$ is the marginal influence of $$v_j$$ according to the greedy selection order. Suppose the landmark set $$\Lambda = \{\lambda ^{c}_0, \lambda ^{c}_1, \lambda ^{c}_2, \ldots , \lambda ^{c}_m\}$$. For every $$\lambda \in \Lambda $$, we pre-compute $$S^{g}(k, \lambda p_i)$$, for every single topic $$i \in [d]$$, and cache $${ MI}^{g}(v, \lambda p_i)$$, $$\forall v \in S^{g}(k, \lambda p_i)$$ in advance by Algorithm 1.

#### *Online stage*

Marginal Influence Sort (MIS) algorithm as described in Algorithm 3. Given an item $I = (\lambda _1, \ldots , \lambda _{d})$, the online processing stage first rounding down the mixture to $I'= (\underline{\lambda }_1, \ldots , \underline{\lambda }_d)$, and then use the union $V^g = \cup _{i \in [d], \underline{\lambda }_i > 0} S^{g}(k, \underline{\lambda }_i p_i)$ as seed candidates. If a node appears in multiple pre-computed seed sets, we add their marginal influence in each set together (line 4). Then we simply sort all nodes in $V^g$ according to their computed marginal influence *f*(*v*) and return the top-*k* nodes as seeds.

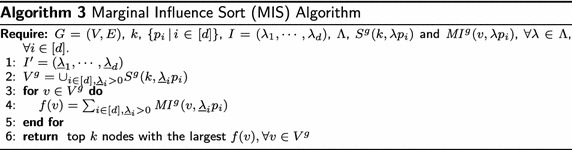



Although MIS is a heuristic algorithm, it does guarantee the same performance as the original greedy algorithm in fully separable networks when the topic mixtures is from the landmark set, as shown by the theorem below. Note that in a fully separable network, it is reasonable to assume that seeds for one topic comes from the subgraph for that topic, and thus seeds from different topics are disjoint.

##### **Theorem 2**

*Suppose* $I = (\lambda _1, \lambda _2, \ldots , \lambda _d)$,* where each* $\lambda _i \in \Lambda$,* and* $S^{g}(k, \lambda _1 p_1)$, $\ldots$, $S^{g}(k, \lambda _d p_d)$ *are disjoint. If the network is fully separable for all topics, the seed set calculated by Algorithm *3* is one of the possible sequences generated by Algorithm* 1* under the mixed influence probability* $p = \sum _{i \in [d]} \lambda _i p_i$.

*Proof sketch* Denote $v_1, v_2, \ldots , v_k \in V^g$ as the final seeds selected for the topic mixture in this order, and let $S_0 = \emptyset$ and $S_\ell = S_{\ell - 1} \cup \{ v_\ell \}$, for $\ell = 1,2,\ldots ,k$. Since the network is fully separable and topic-wise seed sets are disjoint, by Lemma 4.1 we can get that $v_1, v_2, \ldots , v_k$ are selected from topic-wise seeds sets, and $\forall v \in V^g$, $f(v) = { MI}(v | S_{\ell - 1}, p)$. We can prove that $v_{\ell } = \mathop {\arg \max }\nolimits _{v \in V\backslash {S_{l - 1}}}$ ${ MI}(v | S_{\ell -1}, p)$, $\forall \ell = 1,2,\ldots ,k$ by induction. It is straightforward to see that $v_1 = \mathop {\arg \max }\nolimits _{v \in V}$ ${ MI}(v | \emptyset , p)$. Assume it holds for $\ell = j \in \{1,2,\ldots ,k-1\}$. Then, for $\ell = j+1$, for a contradiction we suppose that the $(j+1)$-th seed $v'$ is chosen from $V \setminus V^{g}$ other than $v_{j+1}$, i.e., ${ MI}(v' | S_{j}, p) > { MI}(v_{j+1} | S_{j}, p)$. Denote $i'$ such that $\sigma (v', p_{i'}) > 1$. Since budget $k > j$, we can find a node $u \in S^{g}(k, \lambda _{i'} p_{i'}) \setminus S_{j}$, such that ${ MI}(u | S_{j}, \lambda _{i'} p_{i'})$ $\ge$ ${ MI}(v' | S_{j}, \lambda _{i'} p_{i'})$, and *u* is selected before $v_{j+1}$, which is a contradiction. Therefore, we will conclude that $v_1, v_2$, $\cdots$, $v_k$ is one possible sequence from the greedy algorithm. $\hfill\square$

The theorem suggests that MIS would work well for networks that are fairly separated among different topics, which are verified by our test results on the Arnetminer dataset. Moreover, even for networks that are not well separated, it is reasonable to assume that the marginal influence of nodes in the mixture is related to the sum of its marginal influence in individual topics, and thus we expect MIS to work also competitively in this case, which is verified by our test results on the Flixster dataset.

## Experiments

We test the effectiveness of our algorithms by using a number of real-world datasets, and compare them with state-of-the-art influence maximization algorithms.

### Algorithms for comparison

In our experiments, we test our topic-aware preprocessing based algorithms MIS and BTS comprehensively. We also select three classes of algorithms for comparison: (a) Topic-aware algorithms: The topic-aware greedy algorithm (TA-Greedy) and a state-of-the-art fast heuristic algorithm PMIA (TA-PMIA) from [10]; (b) Topic-oblivious algorithms: The topic-oblivious greedy algorithm (TO-Greedy), degree algorithm (TO-Degree) and random algorithm (Random); (c) Simple heuristic algorithms that do not need preprocessing: The topic-aware PageRank algorithm (TA-PageRank) from [18] and WeightedDegree algorithm (TA-WeightedDegree).

The greedy algorithm we use employs lazy evaluation [5] to provide hundreds of time of speedup to the original Monte Carlo based greedy algorithm [1], and also provides the best theoretical guarantee. PMIA is a fast heuristic algorithm for the IC model based on trimming influence propagation to a tree structure and fast recursive computation on trees, and it achieves thousand fold speedup comparing to optimized greedy approximation algorithms with a small degradation on influence spread [10] (in this paper, we set a small threshold $\theta = 1/1280$ to alleviate the degradation).

Topic-oblivious algorithms work under previous IC model that does not identify topics, i.e., it takes the fixed mixture $\forall j\in [d], \lambda _j=\frac{1}{d}$. TO-Greedy runs greedy algorithm for previous IC model and uses the top-*k* nodes as its seeds. TO-Degree outputs the top-*k* nodes with the largest degree based on the original graph. Random simply chooses *k* nodes at random.

We also carefully choose two simple heuristic algorithms that do not need preprocessing. TA-PageRank uses the probability of the topic mixture as its transfer probability, and runs PageRank algorithm to select *k* nodes with top rankings. The damping factor is set to 0.85. TA-WeightedDegree uses the degrees weighted by the probability from topic mixtures, and selects top *k* nodes with the highest weighted degrees.

Finally, we study the possibility of acceleration for large graphs by comparing PMIA with greedy algorithm in preprocessing stage. Therefore, we denote MIS and BTS algorithms, utilizing the seeds and marginal influence from greedy and PMIA, as MIS[Greedy], BTS[Greedy] and MIS[PMIA], BTS[PMIA], respectively.

### Experiment setup

We conduct all the experiments on a computer with 2.4 GHz Intel(R) Xeon(R) E5530 CPU, 2 processors (16 cores), 48G memory, and an operating system of Windows Server 2008 R2 Enterprise (64 bits). The code is written in C++ and compiled by Visual Studio 2010.

We test these algorithms on the Flixster and Arnetminer datasets as we described in "Data observation" section, which have the advantage that the influence probabilities of all edges on all topics are learned from real action trace data or node topic distribution data. To further test the scalability of different algorithms, we use a larger network data DBLP, which is also used in [10]. DBLP^3^ is an academic collaboration network extracted from the online service, where nodes represent authors and edges represent coauthoring relationships. It contains 650K nodes and 2 million edges. As DBLP does not have influence probabilities from the real data, we simulate two topics according to the joint distribution of topics 1 and 2 in the Flixster and follow the practice of the TRIVALENCY model in [10] to rescale it into 0.1, 0.01, or 0.001, standing for strong, medium, and low influence, respectively.

In terms of topic mixtures, in practice and also supported by our data, an item is usually a mixture of a small number of topics, thus our tests focus on testing topic mixtures from two topics. First, we test random samples to cover most common mixtures as follows. For these three datasets, we use 50 topic mixtures as testing samples.^4^ Each topic mixture is uniformly selected from all possible two topic mixtures. Second, since we have the data of real topic mixtures in Flixster dataset, we also test additional cases following the same sampling technique described in "Data description" section of [17]. We estimate the Dirichlet distribution that maximizes the likelihood over topics learned from the data. After the distribution is learned, we resample 50 topic mixtures for testing.

In the preprocessing stage, we use two algorithms, Greedy and PMIA, to pre-compute seed sets for MIS and BTS, except that for the DBLP dataset, which is too large to run the greedy algorithm, we only run PMIA. Algorithms MIS and BTS need to pre-select landmarks $\Lambda$. In our tests, we use 11 equally distant landmarks $\{0, 0.1, 0.2, \ldots , 0.9, 1\}$. Each landmarks can be pre-computed independently, therefore we run them on 16 cores concurrently in different processes.

We choose $k=50$ seeds in all our tests and compare the influence spread and running time of each algorithm. For the greedy algorithm, we use 10,000 Monte Carlo simulations. We also use 10,000 simulation runs and take the average to obtain the influence spread for each selected seed set.

In addition, we apply offline bound and online bound to estimate influence spread of optimal solutions. Offline bound is the influence spread of any greedy seeds multiplied by factor $1/(1-{\rm e}^{-1})$. The online bound is based on Theorem 4 in [5]: for any seed set *S*, its influence spread plus the sum of top *k* marginal influence spread of *k* other nodes is an upper bound on the optimal *k* seed influence spread. We use the minimum of the upper bounds among the cases of $S=\emptyset$ and *S* being one of the greedy seed sets selected.

### Experiment results

Additional file 1: Figure S1 shows the total influence spread results on Arnetminer with random samples (a); Flixster with random and Dirichlet samples, (b) and (c), respectively; and DBLP with random samples (d). Table 8a shows the preprocessing time based on greedy algorithm and PMIA algorithm on three datasets. Table 8b shows the average online response time of various algorithms in finding 50 seeds (topic-oblivious algorithms always use the same seeds and thus are not reported).

**Table 8:** Running time statistics

	Arnetminer		Flixster		DBLP	
	($|\Lambda |=8\times 11$)		($|\Lambda |=10\times 11$)		($|\Lambda |=2\times 11$)	
	Total	Max	Total	Max	Total	Max
(a) Preprocessing time						
Greedy	8.8 h	1.2 h	26.3 days	3.5 days	≥100 days	≥7 days
PMIA	37 s	7.1 s	2.28 h	12.6 min	9.6 min	4.2 min

	Arnetminer	Flixster		DBLP		
		Random	Dirichlet			
(b) Average online response time						
TA-Greedy	9.3 min	1.5 days	20 h	N/A		
TA-PMIA	0.52 s	5.5 min	3.8 min	58 s		
MIS (µs)	2.85	2.37	3.84	2.09		
BTS (µs)	1.20	2.35	1.42	0.49		
TA-PageRank (s)	0.15	2.08	2.30	41		
TA-WeightedDegree	8.5 ms	29.9 ms	30.7 ms	0.32 s		

As is shown in Table 8a, we run each landmark concurrently, and count both the total CPU time and the maximum time needed for one landmark. While the total time shows the cumulative preprocessing effort, the maximum time shows the latency when we use parallel preprocessing on multiple cores. The results indicate that the greedy algorithm is suitable for small graphs but infeasible for large graphs like DBLP, while PMIA is a scalable preprocessing solution for large graphs. For this reason, we test two preprocessing techniques and also compare their performance.

For the Arnetminer dataset (Additional file 1: Figure S1), it clearly separates all algorithms into three tiers: the top tier is TA-Greedy, TA-PMIA, MIS[Greedy] and MIS[PMIA]; the middle tier is TA-WeightedDegree, BTS[Greedy], BTS[PMIA] and TA-PageRank; and the lower tier is topic-oblivious algorithms TO-Greedy, TO-Degree and Random. In particular, we measure the gaps of influence spread among different algorithms. We observe that the gap of top tiers are negligible, because TA-PMIA, MIS[Greedy] and MIS[PMIA] are only 0.61, 0.32 and $1.08\%$ smaller than TA-Greedy, respectively; the middle tier algorithms BTS[Greedy], BTS[PMIA], TA-WeightedDegree and TA-PageRank are 4.06, 4.68, 4.67 and $26.84\%$ smaller, respectively; and the lower tier TO-Greedy, TO-Degree and Random have difference of 28.57, 56.75 and $81.48\%$, respectively. (All percentages reported in this section are averages over influence spread from one seed to 50 seeds.)

The detailed analyses are listed as follows: First, topic-oblivious algorithms does not perform well in topic-aware environment. Based on Observation 1, when topics are separated, algorithms ignoring topic mixtures cannot find influential seeds for all topics, and thus do not have good influence spread. Second, MIS[Greedy] and MIS[PMIA] almost match the influence spread of those of TA-Greedy and TA-PMIA. As indicated from offline and online bounds, MIS[Greedy], BTS[Greedy] are 76.9 and 72.5% of the online bound, which demonstrates their effectiveness is better than their conservative theoretical bounds ($1-{\rm e}^{-1} \approx 63.2\%$). The MIS algorithm runs noticeably fast in online processing, finishing 50 seeds selection in just a few microseconds (Table 8b), which is three orders of magnitude faster than the millisecond response time reported in [17], and at least three orders of magnitude faster than any other topic-aware algorithms. This is because it relies on pre-computed marginal influence and only a sorting process is needed online. Third, BTS[Greedy] and BTS[PMIA] are not expected to be better than MIS[Greedy] and MIS[PMIA], since BTS is a baseline algorithm only selecting a seed set from one topic. However, due to the preprocessing stage, we find that it can even perform better than other simple topic-aware heuristic algorithms that have short online response time. In addition, replacing the greedy algorithm with PMIA in the preprocessing stage, MIS and BTS only lose 0.76 and $0.62\%$ in influence spread, indicating that PMIA is a viable choice for preprocessing, which greatly reduces the offline preprocessing time (Table 8a).

What we can conclude from tests on Arnetminer is that, for networks where topics are well separated among nodes and edges such as in academic networks, utilizing preprocessing can greatly save the online processing time. In particular, MIS algorithm is well suited for this environment achieving microsecond response time with very small degradation in seed quality.

For Flixster dataset (Additional file 1: Figure S1), we see that the influence spread of TA-PMIA, MIS[Greedy], MIS[PMIA], BTS[Greedy] and BTS[PMIA] are 1.78, 3.04, 4.58, 3.89 and $5.29\%$ smaller than TA-Greedy for random samples, and 1.41, 1.94, 3.37, 2.31 and $3.59\%$ smaller for Dirichlet samples, respectively. In Flixster, we can see that for networks where topics overlap with one another on nodes, our preprocessing based algorithms can still perform quite well. This is because most seeds of topic mixtures are from the constituent topics (Observation 2). On the other hand, the influence of TA-WeightedDegree, TA-PageRank and TO-Greedy will suffer a noticeable degeneration demonstrated from two curves. In terms of online response time (Table 8b), the result is consistent with the result for Arnetminer: only MIS and BTS can achieve microsecond level online response, and all other topic-aware algorithms need at least milliseconds since they at least need a ranking computation among all nodes in the graph. In addition, TA-PMIA on Flixster is much slower than on Arnetminer, because both the network size and the computed MIA tree size are much larger, indicating that PMIA is not suitable in providing stable online response time. In contrast, the response time of MIS and BTS do not change significantly among different graphs.

In DBLP (Additional file 1: Figure S1), the graph is too large to run greedy algorithm, thus we take TA-PMIA as the baseline algorithm to compare with other algorithms. For different algorithms, the influence spread is close to each other, and our results show that MIS[PMIA] has equal competitive influence spread with TA-PMIA ($0.44\%$ slightly larger), while BTS[PMIA], TA-WeightedDegree, TO-Degree and TA-PageRank are 1.33, 1.83, 6.05 and $35.54\%$ smaller than TA-PMIA, respectively. Combining the running time in Table 8, we find that the greedy algorithm is not suitable for preprocessing for large graphs, while PMIA can be used in this case.

To summarize, the greedy algorithm has the best influence spread performance, but is slow and not suitable for large-scale networks or fast response time requirements. PMIA as a fast heuristic can achieve reasonable performance in both influence spread and online processing time, but its response time varies significantly depending on graph size and influence probability parameters, and could take minutes or longer to complete. Our proposed MIS emerges as a strong candidate for fast real-time processing of topic-aware influence maximization task: it achieves microsecond response time, which does not depend on graph size or influence probability parameters, while its influence spread matches or is very close to the best greedy algorithm and outperforms other simple heuristics. Furthermore, in large graphs where greedy is too slow to finish, PMIA is a viable choice for preprocessing, and our MIS using PMIA as the preprocessing algorithm achieves almost the same influence spread as MIS using the greedy algorithm for preprocessing.

## Related work

Domingos and Richardson [3, 4] are the first to study influence maximization in an algorithmic framework. Kempe et al. [1] first formulate the discrete influence diffusion models including the independent cascade model and linear threshold model, and provide the first batch of algorithmic results on influence maximization.

A large body of work follows the framework of [1]. One line of research improves on the efficiency and scalability of influence maximization algorithms [9–11, 19]. Others extend the diffusion models and study other related optimization problems [6, 7, 12]. A number of studies propose machine learning methods to learn influence models and parameters [13–15]. A few studies look into the interplay of social influence and topic distributions [14, 20–22]. They focus on inference of social influence from topic distributions or joint inference of influence diffusion and topic distributions. They do not provide a dynamic topic-aware influence diffusion model nor study the influence maximization problem. Barbieri et al. [16] introduce the topic-aware influence diffusion models TIC and TLT as extensions to the IC and LT models. They provide maximum-likelihood based learning method to learn influence parameters in these topic-aware models. We use the their proposed models and their datasets with the learned parameters.

A recent independent work by Aslay et al. [17] is the closest one to our work. Their work focuses on index building in the query space while we use pre-computed marginal influence to help guiding seed selection, and thus the two approaches are complementary. Other differences have been listed in the introduction and will not be repeated here.

## Future work

One possible follow-up work is to combine the advantages of our approach and the approach in [17] to further improve the performance. Another direction is to study fast algorithms with stronger theoretical guarantee. An important work is to gather more real-world datasets and conduct a thorough investigation on the topic-wise influence properties of different networks, similar to our preliminary investigation on Arnetminer and Flixster datasets. This could bring more insights to the interplay between topic distributions and influence diffusion, which could guide future algorithm design.

## Additional file

**Additional file 1: Figure S1.** Influence spread of algorithms. Subfigures: (**a**) Arnetminer on random samples; (**b**) Flixster on random samples; (**c**) Flixster on Dirichlet samples; (**d**) DBLP on random samples. Legends are ordered (left to right, top to bottom) according to influence spread.

## Footnotes

1
http://www.flixster.com.

2 arnetminer.org.

3
http://www.DBLP.org.

4 50 samples is mainly to fit for the slow greedy algorithm.
