# Supplementary material for: Real-time topic-aware influence maximization using preprocessing
Source: Comput Soc Netw. 2016 Nov 10;3(1):8. doi: 10.1186/s40649-016-0033-z (PMC5748872; doi:10.1186/s40649-016-0033-z)
Supplement: Supplementary file 1 — Additional file 1: Figure S1. Influence spread of algorithms. Subfigures: (a) Arnetminer on random samples; (b) Flixster on random samples; (c) Flixster on Dirichlet samples; (d) DBLP on random samples. Legends are ordered (left to right, top to bottom) according to influence spread. [file 40649_2016_33_MOESM1_ESM.pdf]

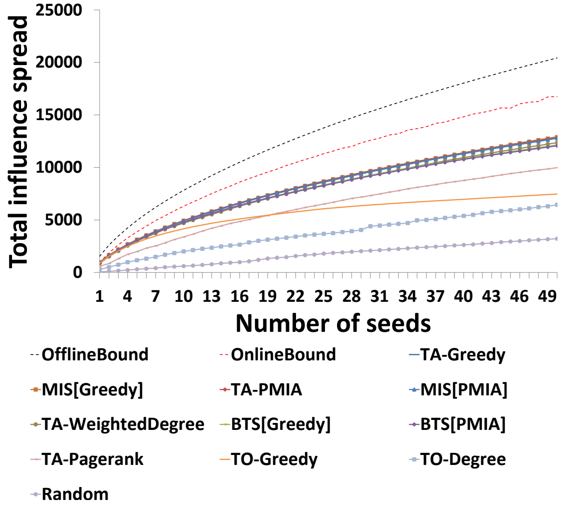

(a) Arnetminer on random samples

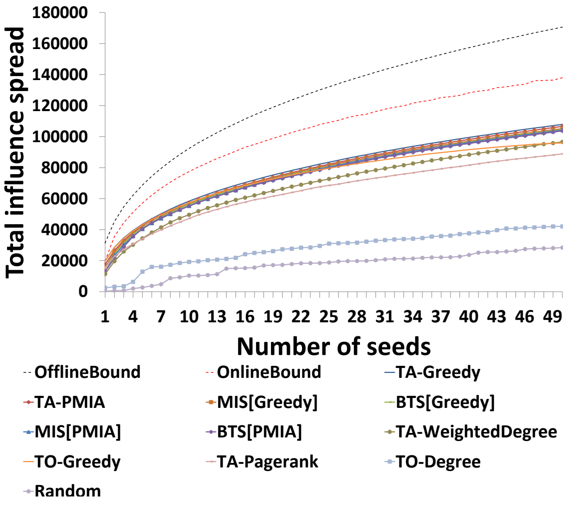

(b) Flixster on random samples

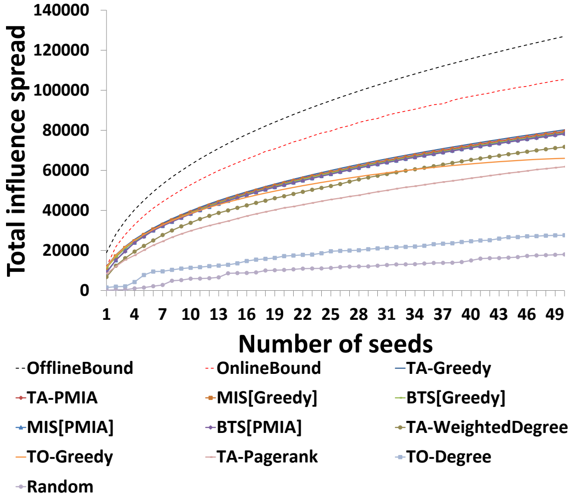

(c) Flixster on Dirichlet samples

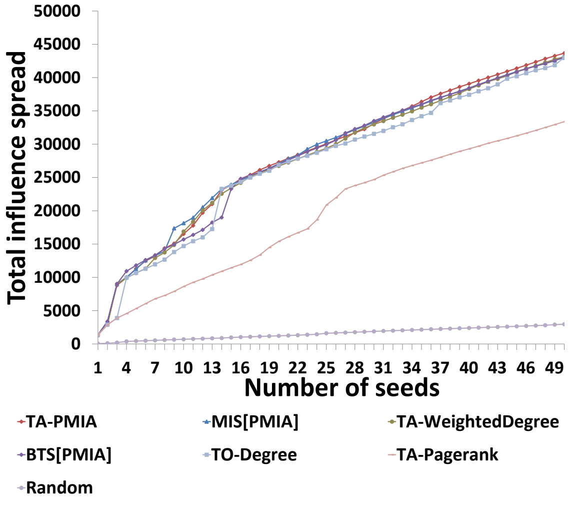

(d) DBLP on random samples
